# Short Notes of Post-Mortem Examinations

**Published:** 1871-11

**Authors:** 


### Short Notes of Post-Mortem Examinations.—Vienna.

For these notes, and the following case reported from the Vienna General Hospital, we are indebted to Mr. Edward Sparks.

*Primary Cancer of the Lungs.*—A woman, aged twenty-two, died, as was supposed, from pleuro-pneumonia. On opening the throax, the right pleura was found to contain some false membrane, in which were imbedded several soft greyish-white nodules of encephaloid cancer. Similar small nodules existed in the lower lobe of the right lung, the apex of which contained a small bronchopneumonic cavity and some caseous infiltration of similar origin. The left upper lobe contained no cancer nodules, but was intensely congested towards the apex, and lower down was hepatised. The upper part of the lower left lobe was also free from cancer, but the lower two-thirds consisted of a huge mass of encephaloid nodules, one of which was as large as a man's fist. The growth extended towards the mid-line of the body, pushing the heart far over to the right. It projected also into the left pleura, and had infiltrated the diaphragm, so that nodules projected on its under side. No cancer was found in the abdominal organs, nor in any other part of the body except the thorax.

*Caries of Vertebræ; abscess opening into the spinal canal; no paralysis.*—In this case, caries of the bodies of the third and fourth lumbar vertebræ and destruction of the intervening fibro-cartilage led, on the one side to a large psoas abscess, and, on the other, to perforation of the spinal canal and entrance of pus into the latter. Although at the post-mortem the canal was found to be filled with pus below the fourth lumbar vertebra, so that the cauda equina was bathed in it, yet the man had not a trace of paralysis during his illness, and no implication of the spinal cord was even suspected.

*Intra-pericardial Rupture of the Aorta in a boy of sixteen.*—The subject of the necropsy died suddenly while at dinner. He was only sixteen years old, and was supposed to have been previously healthy. On opening the pericardium, a large loose blood-clot of the size of two fists was found distending it. The heart had a hypertrophied left ventricle, whose wall was an inch thick. The cavity was but slightly dilated. The mitral and aortic valves were healthy. Just above the aortic valves was found a small irregular rent in the adjacent aortic wall—all but over a patch of advanced atheromatous degeneration. The intima was slightly saculated at this point. On the pericardial side of the aorta existed a larger tear, through which the blood had made its way into the pericardium. On closer examination it was found that at the point where the aorta was penetrated a dissecting aneurism had been formed. The blood, after rupturing the intima, had split the media into two parts, so that one-half remained adherent to the intima and the other to the adventitia; finally, it had broken through the latter also. This case is interesting from the early age of the patient at which atheroma led to a fatal issue.

*Disease of Tricuspid and Pulmonary Artery Valves, without Affection of the left heart.*—The patient, a man of twenty-seven, died suddenly in a state of extreme cyanosis. There was no history of any previous illness. On opening the pericardium, the heart appeared quite round from distension of the right side with blood. The mitral and aortic valves and the left cavities were found perfectly healthy. On the right side, however, the ventricle was so dilated and thinned that its wall appeared to be made up almost entirely of endocardium and peri-cardium, through atrophy of its muscular substance. The endocardium was opaque and thickened. The tricuspid valve was insufficient, its segments thickened, and its free borders beaded from endocarditis. The pulmonary artery was very narrow where it left the ventricle, with valves shrunken up to its walls, and adherent by their edges, so that the orifice could not be closed at all. This was probably a case of congenital stenosis of the pulmonary artery, followed by disease of the tricuspid and ventricle later on, just as in another congenital abnormity of the heart—transposition of the vessels—the transposed aorta becomes the subject of endocarditis.—*Lancet.*

